# A Communication Toolkit to Assist Families Access Dental Care Services in Scotland: A Qualitative Evaluation †

**DOI:** 10.3390/dj13020080

**Published:** 2025-02-14

**Authors:** Sucharita Nanjappa, Thushani I. D. Wijesiri, Susan J. Carson, Ruth Freeman

**Affiliations:** 1 Dental Public Health, School of Dentistry, University of Dundee, 2 Park Pl, Dundee DD1 4HR, UK; 2 Public Health, NHS Forth Valley, Carseview House, Castle Business Park, Stirling FK9 4SW, UK

**Keywords:** communication tool, evaluation, dental appointments, preventive visits

## Abstract

**Background:** Childsmile is Scotland’s national child oral health improvement programme designed to reduce inequalities in oral health and ensure universal access to NHS dental services by working closely with children and their families. Research has shown that parents with more dental concerns are less likely to bring their children for regular preventive dental check-ups.Therefore, a communication toolkit named ‘Chatterbox’ was created to help families access dental care services. **Methods:** This study aimed to evaluate the acceptability of Chatterbox from the perspective of parents with young children and from staff who work with them. Thirty parents of young children, volunteers, and staff affiliated with three third-sector family support organisations in Dundee participated in in-depth interviews, focus groups, and observations to assess the objectives of Chatterbox. A framework analysis was conducted to capture the themes, patterns, and findings triangulated using observations and field notes. **Results:** Chatterbox was found to promote effective communication, identify barriers to dental attendance, provide a platform for reflection, convey the complexities of daily life, and boost parents’ confidence to seek assistance. **Conclusions:** Chatterbox has the potential to help parents in accessing preventive dental care for their young children when used with trained support workers.

## 1. Introduction

The introduction of the Childsmile Programme has been credited with the advancements made in the oral health of Scottish children in recent years [1,2]. Childsmile is a national oral health initiative in Scotland that aims to improve the oral health of children [1]. The programme has a particular emphasis on delivering interventions in multiple settings, such as nurseries, schools, and primary care dental practice, in order to address oral health inequalities [3,4]. One of the key components of the Childsmile Programme is the development of initiatives to encourage attendance at dental practices for preventative visits, formerly known as ‘Childsmile Practice’ [4].

During the pilot phase of Childsmile Practice, it was found that 32% of the families who enrolled did not attend their planned dental appointments [5]. Notably, a majority of those who failed to attend were living in areas characterised by high levels of relative deprivation [3]. Based on the evaluation of data from 2014/2015, it was found that dental practice attendance remained static at around 70.5% among children aged 4–6 years [6]. Moreover, it was observed that the likelihood of experiencing dental caries decreases with an increase in the number of visits a child makes to primary care dental practice [6]. Additionally, the review confirmed that the visit patterns of children to primary care dental practices were consistent across all the strata of the Scottish Index of Multiple Deprivation (SIMD) [6]. It was found in addition that looked after children, that is those in the care of their local authority, were more likely to have dental treatment needs at five years and less likely to access dental services [7].

There has been minimal research as to why families fail to attend primary dental care appointments. Two previously published qualitative studies identified a lack of transport and childcare, negative perceptions of dental care providers, a lack of parental tradition of going to the dentist, and a lack of parental confidence as barriers to parents attending dental appointments with their children [8,9]. A study conducted in 2016 found that the top three reasons cited by parents for failing to bring their children to appointments were forgetfulness, considering that the appointment was no longer required, and childhood illness [7]. This study highlighted that improving engagement with parents before scheduling appointments reduced perceived barriers to attendance [7]. Factors included in reducing non-attendance were optimising dental clinic efficiency with simultaneous overlapping appointments for new patients, reducing the time between referral and booked appointment, collecting more patient information, repeated reminders, and introducing extended clinic hours [10]. Similar barriers were highlighted in the first and second phases of the Childsmile-funded ‘Developing an inventory to Assess Parental concerns and Enable child dental Registration’ (DAPER) project, where parents with high levels of dental concerns were less likely to engage in preventive dental visits for their children [5,11].

The third phase of the DAPER project saw the creation of a communication toolkit called ‘Chatterbox’ designed to enhance the relationship between families and dental health support workers (DHSWs) and to customise assistance for individual families’ needs. A recent review has highlighted the value and utility of design-based approaches in oral health, which is recognised as being beneficial in addressing complex problems in healthcare [12]. A pilot study involving nine non-attending families was conducted to test Chatterbox [12]. Of the nine, six families successfully attended practice and received dental care after using the toolkit [12]. Qualitative interviews with DHSWs revealed that Chatterbox effectively customised support for families and helped them develop solutions to enhance their likelihood of accessing dental care [10]. The pilot study also found that organisational challenges such as staff shortages and individual factors such as the capability and motivation of the DHSWs reduced their own engagement with Chatterbox [13]. Building on the findings from the pilot study, the next step was to investigate parents’ perspectives on Chatterbox to determine if it could enhance their communication with dental health support workers and improve their children’s access to dental care.

Therefore, the aim of this study was to evaluate the acceptability of Chatterbox from the perspective of parents with young children and from staff who work with them to explore its role in supporting parents’ access to dental care for their children. A second aim was to explore participants’ ideas for modifying Chatterbox, with a view to inform the future development and implementation of the intervention within the Childsmile programme [14].

## 2. Materials and Methods

A qualitative research design of in-depth interviews, focus groups, and observations of parents’ engagement with Chatterbox was used to explore the study’s aim.

The study aimed to recruit a varied group of participants with diverse experiences, including disadvantaged populations who are less likely to attend consultations, such as migrant families. Therefore, family support organisations from the third sector in Dundee were intentionally selected for the study. The following organisations were identified and approached: Home-Start Dundee is an organisation that provides tailored support to local families with at least one child under the age of five; Toddlers & Co is a parent–toddler group organised by Logie and St John’s (Cross) Church of Scotland as a non-faith-based activity for families with toddlers, from Dundee and the surrounding areas, to meet and socialise; and the International Families Group, which is run for the families of international staff and students at the University of Dundee and is run by volunteers. Interested participants were given participant information sheets and written consent was obtained before commencing the study.

The study included the parents of young children who visited these three third-sector family support organisations in Dundee, as well as volunteers and staff working within them. These two target groups were selected to evaluate the toolkit from the perspectives of both service providers and service users. The participants were purposefully chosen to represent cultural diversity, varied socioeconomic backgrounds, and other factors, aiming to capture a comprehensive understanding of the toolkit’s acceptability among diverse groups. A convenient sampling technique was used to recruit the participants.

**Figure 1:**
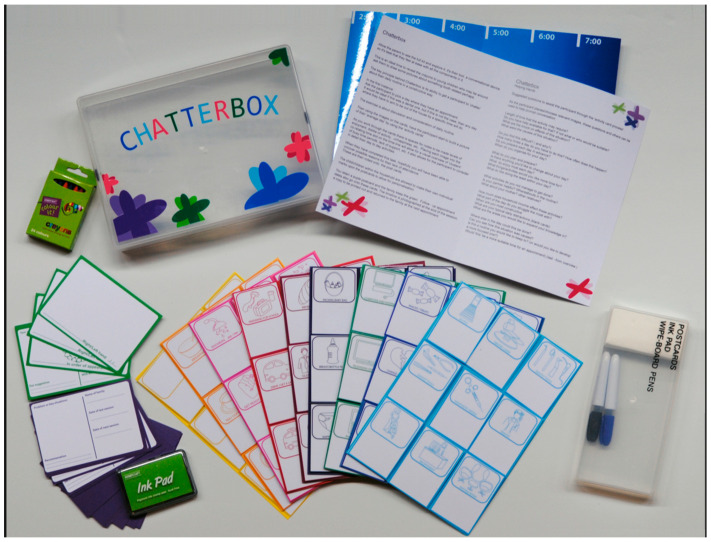
Chatterbox communication toolkit.

The Chatterbox toolkit included a timeline base, 81 reusable activity cards, and appointment postcards. Seventy-two activity cards were colour-coded and categorised, featuring the pictorial representations of everyday activities that families typically engage in, as well as the factors that could impact their dental attendance patterns. Additionally, nine blank cards were included to allow the participants to customise the toolkit as per their own needs (Figure 1).

The participants were prompted to choose activity cards pertinent to their daily routine and arrange them on a timeline base to create a visual representation of a typical day for their family. The activity cards also prompted discussions around potential challenges that might hinder dental attendance, such as transportation, childcare, availability of social support for the family, prior experiences with dental services, and other worries or concerns. The participants were provided with easy wipe markers to annotate the activity cards with comments or notes. The box also contained appointment cards that could be personalised to the family by having the child’s hand or footprint stamped on it. After engaging with the materials in Chatterbox and sharing their thoughts out loud, the participants were further encouraged to express their opinions about the toolkit through in-depth interviews and focus group discussions. Seven different focus groups were carried out based on participant convenience and grouped according to common characteristics (Table 1). The objective of these discussions was to evaluate the acceptability of Chatterbox to them and to explore its role in supporting parents’ access to dental care for their children. A second objective was to explore the participants’ ideas for modifying Chatterbox, with a view to inform the future development and implementation of the intervention within the Childsmile programme These discussions were, in addition, to validate concerns raised in earlier focus groups with the DHSWs around the tool’s acceptability to users [13]. Audio recordings were made of all the interviews and focus groups, and the researcher maintained a diary of her observations. The triangulation of these observations, notes made during and after the interviews, and audio recordings provided a valuable context for the investigation, resulting in a more comprehensive and robust evaluation [15]. Data collection was concluded once no new findings emerged from new sources, and saturation was achieved.

The interviews were transcribed and analysed using framework analysis, a suitable approach to interpreting and elucidating topics pertaining to a specific context [16,17]. The researchers followed all five steps of the framework analysis, including familiarisation, identifying a thematic framework, indexing, charting, and mapping and interpretation, to obtain the results [16,17]. Ethical approval was obtained from the University of Dundee Research Ethics Committee (UREC 15214). Participation was voluntary, and informed consent was obtained and confidentiality and anonymity were assured and maintained.

## 3. Results

The study comprised 30 participants, including 20 parents or caregivers of young children under five and 10 staff members/volunteers who worked with the parents of young children. Most participants were either born or long settled in Scotland, while six had recently migrated from other countries. The results are presented according to the themes generated through thematic analysis. A description of the participant characteristics is provided in Table 1.

### 3.1. Acceptability of the Chatterbox Toolkit

Overall, the participants were positive in their views of the Chatterbox toolkit. Many thought it was a ‘*brilliant idea*’. They could see that its application went beyond dentistry.

*“I think it’s a great idea, a great little box; like when you pulled it out, I thought, I don’t need that. I can make it to the dentist, but I actually need it … I think it makes a difference in more ways than you think”.* Parent 3.

*“… it certainly seems to be a very good thing which could be used for far more than just the dental health aspect; you could use it for helping family review their days and plan ahead”.* Staff 1.

### 3.2. The Role of Chatterbox in Supporting Families of Young Children Access Dental Care

#### 3.2.1. Facilitate Communication with the DHSWs 

The participants agreed that Chatterbox would encourage conversation with DHSWs. The participants felt Chatterbox could help put parents at ease if they were nervous and that holding the activity cards and populating the timeline took the focus away from them and allowed them to talk more openly without making eye contact.

*“It gives you something to do rather than to speak one-on-one a wee bit awkwardly. Especially if it’s your first-time meeting or even your second time meeting”.* Parent 1

The activity cards were perceived by the participants as useful prompts to initiate conversations, making them feel more comfortable discussing their emotions. However, some participants believed that their willingness to open up would depend on how amiable the facilitator was. If the facilitator was someone they felt at ease with, the participants were happy to share their thoughts candidly. Consequently, the participants saw Chatterbox as a tool that would enable them to communicate with a trusted facilitator rather than to improve the facilitator’s abilities or traits.

*“Obviously, it depends who the support worker is. Somebody who is super non-judgemental and really supportive and maybe somebody who on purpose normalises that it might not be that easy, do you know what I mean, …So, I think who the support worker is will make a big difference to whether people open up or not”.* Parent 2

On the other hand, some participants felt that Chatterbox’s usefulness was not dependent on their relationship with the facilitator; instead, the timeline was inherently valuable to them in organising their day.

*“At the end of the day, it sorts your head out, doesn’t it? It plans you up for the day. So, it doesn’t really matter what anybody else thinks”.* Parent 1

#### 3.2.2. Identification and Discussion of Barriers to Access

According to the participants, the activity cards in Chatterbox were helpful in enabling parents to recognise barriers to attendance that they may not have otherwise identified. Moreover, they prompted conversations between parents and DHSW about issues that may not have been discussed otherwise, such as the absence of social support. The effectiveness of the activity cards in facilitating the identification and communication of barriers to care is further exemplified by the following quotes.

*“… you might not know what the problem is, but then if you look at the cards, it kind of highlights, oh actually, that is the problem!”.* Parent 3

*“It’s always good having something visual, having props to be able to start a conversation. I think that’s a really useful thing”*… *“you can see and it will remind you”.* Staff 1

In addition, the participants observed that Chatterbox could prompt them to ask for help. The communication tool could help overcome the misconception that they had to manage without asking for help or that their parenting skills would be judged as lacking if they asked for help.

*“As a mum, I think it’s really difficult to admit when you’re kind of [struggling] because you feel like you might be failing or something like that, so it’s difficult”.* Parent 5

Chatterbox could also help identify some hidden sensitive issues that hinder access to care. For example, letting their DHSW know that they need assistance to fill out forms at the dentist because of limited literacy skills.

*“Not many words on it either, which is good because that can be quite daunting. So least this, like doesn’t have a lot of words and things like that and maybe it would make it easier to come up to the support visitor, that you know, that is one of the issues… they are worried about is kind of filling out forms”.* Parent 2

The participants whose first language was not English emphasised the usefulness of Chatterbox for parents who might have difficulties communicating in English. They found that Chatterbox was particularly helpful in enabling them to communicate their dental concerns, given their limited English language proficiency.

*“… for someone with not much English they might be quite helpful”.* Parent 13

Chatterbox allowed the participants to discuss their perception of oral healthcare delivery openly. They voiced their views on how they perceived the risk from dental diseases to be low for their children but that their perceived cost of the dental visit was high.

*“… this is too much hard work; with a baby that’s got hardly any teeth, I don’t need to bring this kid to the dentist”.* Parent 15

By engaging with Chatterbox, the participants were able to discuss their own dental experiences and became more interested in exploring the factors that might affect their ability to take their children to the dentist. They were also able to ask questions about tooth-brushing techniques for their children, the appropriate age for their first dental check-up, the recommended frequency of visits, and other related concerns that they might have been unsure about.

Additionally, Chatterbox encouraged the participants to communicate their worries about child dental visits, such as the possibility of arriving late and facing consequences like fines or disapproval. Many participants felt they were in a difficult situation where they had to leave their house early to account for any delays but then had to endure longer waiting times at the dentist’s office, which was not seen as very family-friendly and made it challenging to keep their child entertained and content.

#### 3.2.3. Reflection on Daily Activities

Chatterbox helped the parents reflect on how they spent their time during the day, organise their days efficiently, and identify the most convenient times for dental visits.

*“I think it’s good to be able to think about your day; sometimes you don’t realise when you are losing time and when you are really busy. It’s good to think about what kind of routine you have and find out what you can change”.* Parent 5

*“Well, actually, between two and three, I don’t do anything on a daily basis so I could book my dentist appointment for between two and three”.* Parent 1

The participants felt that the timeline and activity cards made discussing and reflecting in depth on their day easier.

*“But that is more tasks and activities like dressing the children or preparing a meal. It’s not just “I have to be here, and I have to be there”. It’s actually getting them to think about how they’re using their time, which I think is helpful for some parents who, as you say, the day just washes over them”.* Staff 1

The participants felt that a tool to help families reflect on their day would allow the parents to identify their problems, which is the first step to successfully organising dental attendance.

*“I think it would help the family identify themselves the issues. They’ll say “look, we’re doing too much in the morning*; *we need to spread that out through the day”.* Staff 1

*“The power in tools like this is it’s not saying “this is your problem”. It’s helping people to work out themselves, and that’s where change comes best when people see it themselves”.* Staff 1

### 3.3. Design of Chatterbox

All the mothers liked being able to touch, hold, and interact with the various elements of Chatterbox; only the participating father said he would prefer a digital version. The suggestion of making a digital version was vetoed by the other participants, who felt this would stifle conversation.

Personalised appointment cards were considered a good idea by all; it was thought to be *“more personal… something that would go in the keepsake box which held childhood memories”.* Parent 10

*“I like the hand with the ink on their appointment cards. Because I think if children were to do that and it was on their fridge, they’d remind their parents as well because they see something that they created with their own hand, and it’s interactive as well”.* Staff 1

Several participants provided feedback on how to improve the physical properties of Chatterbox. Some suggested making it smaller, about the size of an A4 sheet, as they found it too bulky, while others disagreed and were content with its current size. Another participant suggested incorporating a magnet strip to attach the activity cards, as using blue tack putty was deemed “too fiddly”. Adding a handle or placing the kit in a bag for easier transportation was also suggested. One participant proposed including a game within the kit that would allow children to ask questions about dental care or dental visits. This individual believed that if children could understand the importance of brushing twice a day, they would encourage their parents to follow through. Lastly, one participant recommended creating a version of the timeline that could serve as a planner and be hung on the wall.

Following the interview, a participant expressed that the design flaws were insignificant and could be readily rectified, but the overall concept of Chatterbox was remarkable, and she would welcome a visit from someone with it in her home. She believed that the possible uses of Chatterbox far surpassed its design shortcomings.

The above findings suggest that Chatterbox could help parents identify and overcome problems that contribute to missing dental appointments and is helpful in building good relationships with DHSWs.

## 4. Discussion

Chatterbox functions as a communication tool designed to foster meaningful dialogue through interactive patient prompts. It offers patients the opportunity to identify their needs and express concerns or anxieties prior to appointments, potentially reducing missed appointments and enhancing various aspects of oral health-related quality of life over time. The primary aim of this study was to assess how patients perceive Chatterbox and its potential role in aiding parents in accessing dental care for their children. Comparably, in healthcare literature, Patient Concerns Inventories (PCIs) are widely employed to facilitate significant discussions between healthcare providers and patients, particularly in challenging environments such as head and neck cancer clinics [18]. These tools empower patients to voice concerns that might otherwise go unaddressed [19], leading to improvements in patient satisfaction [20] and the overall quality of life [21].

Overall, the study participants were very accepting of Chatterbox. They felt it was useful in bringing to the forefront issues that stopped them from taking their children to the dentist for preventive services. The study participants who work with families also expressed their willingness to recommend Chatterbox to others in similar roles and would welcome having access to it. The tool’s potential to be used in various other settings, such as schools, community support groups, and third-sector organisations, was also highlighted. It was found that Chatterbox could provide insight into the daily lives of families struggling with time management or difficulty expressing their fears and concerns. Furthermore, Chatterbox could help start a dental conversation within a broader discussion about a family’s hectic schedule. Therefore, further studies exploring Chatterbox’s utility in other settings, where families can be supported to engage more meaningfully with their healthcare providers, is recommended.

Approximately one-third of a cohort of 35,236 children required additional support from a DHSW to connect them to primary dental care services [22]. The results of the Simons et al. study showed that engagement with parents before scheduling appointments, to reduce perceived barriers, could potentially help reduce non-attendance [10]. Tools such as Chatterbox will allow tailored engagement with parents to identify and address barriers to attendance, thereby maximising the support that DHSWs could provide to parents. Full engagement with Chatterbox to identify and address parents’ perceived barriers requires a reasonable time window, which has implications for its implementation. A limitation of the tool may be that it requires a greater investment in terms of time. However, the findings indicate that this has the potential to help families that are struggling, and who are also the most likely to be the ones in most need of oral health services.

Although more confident participants could see value in using Chatterbox regardless of the facilitator, parents are more likely to accept Chatterbox if they feel comfortable with the facilitator, as highlighted by De La Cuesta who found that health visitors are required to have excellent communication skills to encourage honesty in interactions with parents [23]. Therefore, based on the findings from this study, training facilitators such as DHSWs will maximise the effectiveness of toolkits such as Chatterbox, which has the potential to positively impact the future dental health and attendance patterns of children, by creating a positive experience for both parents and children.

## 5. Conclusions

Chatterbox has the potential to help parents access dental care services for their children by facilitating effective communication, empowering them to recognise the barriers they may face when attending dental appointments, providing them with a platform to discuss the daily challenges they encounter, and boosting their confidence in seeking help. DHSWs may utilise Chatterbox in various ways such as using it to establish new relationships with families or assisting families they have already built rapport with to identify and discuss issues around attendance and develop strategies to overcome them.

## Figures and Tables

**Table 1:** Description of participants involved in the Chatterbox evaluation by organisation.

Organisation	Number
Home start mothers (Interviewed as two separate focus groups consisting of four and five participants on two separate occasions)	9
International Families Group (Two mothers + two grandmothers/carers interviewed as one group)	4
Toddler & Co (six mothers + one father interviewed in three groups of three, two, and two)	7
Home-Start Dundee volunteers working with young families (interviewed as one group)	10
Total number of participants	30

## Data Availability

The datasets presented in this article are not readily available because of potential for actual or deductive disclosure of participant identity.
